# Countering impaired glucose homeostasis during catch-up growth with essential polyunsaturated fatty acids: is there a major role for improved insulin sensitivity?

**DOI:** 10.1038/s41387-020-00143-y

**Published:** 2021-01-07

**Authors:** Julie Calonne, Helena Marcelino, Christelle Veyrat-Durebex, Isabelle Scerri, Abdul G. Dulloo

**Affiliations:** 1 Department of Endocrinology, Metabolism & Cardiovascular system, University of Fribourg, Fribourg, Switzerland; 2 Department of Chemistry, University of Beira Interior, Covilhã, Portugal; 3 CICS-UBI Health Sciences Research Center, University of Beira Interior, Covilhã, Portugal; 4 Department of Cell Physiology and Metabolism, Faculty of Medicine, Diabetes Center of the Faculty of Medicine, University of Geneva, 1211 Geneva, Switzerland

**Keywords:** Medical research, Diseases

## Abstract

**Background/Objectives:**

Catch-up growth, an important risk factor for later obesity and type 2 diabetes, is often characterized by a high rate of fat deposition associated with hyperinsulinemia and glucose intolerance. We tested here the hypothesis that refeeding on a high-fat diet rich in essential polyunsaturated fatty acids (ePUFA) improves glucose homeostasis primarily by enhancing insulin sensitivity in skeletal muscles and adipose tissues.

**Methods:**

Rats were caloric restricted for 2 weeks followed by 1–2 weeks of isocaloric refeeding on either a low-fat (LF) diet, a high-fat (HF) diet based on animal fat and high in saturated and monounsaturated fatty acids (HF SMFA diet), or a HF diet based on vegetable oils (1:1 mixture of safflower and linseed oils) and rich in the essential fatty acids linoleic and α-linolenic acids (HF ePUFA diet). In addition to measuring body composition and a test of glucose tolerance, insulin sensitivity was assessed during hyperinsulinemic-euglycemic clamps at the whole-body level and in individual skeletal muscles and adipose tissue depots.

**Results:**

Compared to animals refed the LF diet, those refed the HF-SMFA diet showed a higher rate of fat deposition, higher plasma insulin and glucose responses during the test of glucose tolerance, and markedly lower insulin-stimulated glucose utilization at the whole body level (by a-third to a-half) and in adipose tissue depots (by 2–5 folds) during insulin clamps. While refeeding on the ePUFA diet prevented the increases in fat mass and in plasma insulin and glucose, the results of insulin clamps revealed that insulin-stimulated glucose utilization was not increased in skeletal muscles and only marginally higher in adipose tissues and at the whole-body level.

**Conclusions:**

These results suggest only a minor role for enhanced insulin sensitivity in the mechanisms by which diets high in ePUFA improves glucose homeostasis during catch-up growth.

## Introduction

Catch-up growth has long been considered as an essential feature of recovery from the deleterious effects of perturbed growth on development and health. Since the turn of this millennium, the analysis of large epidemiological databases and clinical studies have suggested that catch-up growth is also a risk factor for the development of type 2 diabetes and cardiovascular diseases later in life^1–5^. While the mechanisms linking catch-up growth to the pathogenesis of these chronic diseases remain elusive, the dynamic state of catch-up growth is often characterized by a disproportionately greater rate of body fat recovery relative to that of lean tissue, with hyperinsulinemia as an early feature of this ‘preferential catch-up fat’ phenomenon^6^.

Using a rat model of semistarvation-refeeding which exhibits preferential catch-up fat associated with hyperinsulinaemia even in the absence of hyperphagia, we previously showed that despite insulin resistance in skeletal muscle, the animals refed on a low-fat diet nonetheless achieved blood glucose homeostasis in that they show normal plasma glucose similar to those of the controls whether in the post absorptive state or in response to a glucose load^7,8^. This is achieved by an increase in insulin-stimulated glucose uptake in adipose tissues which, together with an enhancement in de novo lipogenesis, provide a quantitative important glucose sink^8^. Refeeding on a typical ‘western’ diet high in saturated fat, however, blunts the increased sensitivity of adipose tissues to insulin-stimulated glucose uptake, as well as its capacity for de novo lipogenesis^9^, so that dietary fat offsets the ability of adipose tissue to buffer against glucose spared as a result of skeletal muscle insulin resistance, leading to exacerbated hyperinsulinemia and glucose intolerance.

In the search for dietary fat types that may counter such impaired glucose homeostasis and excessive fat deposition during catch-up fat, previous work in our laboratory^10,11^ involving refeeding isocaloric amounts of high-fat diets varying in fatty acid composition, have revealed major differences among the effects of fats derived from various sources of animals’ or plants’ origin on the recovery of fat and lean mass. Of particular interest was the demonstration that refeeding with diet enriched with oils rich in essential polyunsaturated fatty acids (ePUFA)—linoleic acid and/or α-linolenic acid—prevented the excessive fat deposition and exacerbation of hyperinsulinemia and impaired glucose homeostasis observed during catch-up fat on high-fat diets high in saturated and monounsaturated fatty acids^11,12^.

In order to gain insights into mechanisms by which the high ePUFA diet improves glucose homeostasis during catch-up fat, we investigated here the impact of the high ePUFA diet specifically on glucose metabolism in skeletal muscle and adipose tissue. In particular, we tested the hypothesis here that refeeding on the high ePUFA diet would increase insulin sensitivity in both skeletal muscle and adipose tissues by measuring the in vivo glucose utilization in these tissues during hyperinsulinemic-euglycemic clamps, associated with the labeled 2-deoxy-glucose technique.

## Materials and methods

### Animals

Sprague-Dawley rats (Elevage Janvier, Le genest Saint Isle, France), 6 weeks old males, were adapted to room and cage environments for at least 5 days prior to the start of each experiment. They were caged singly in a controlled room (22 ± 1 °C) with a 12-h light–dark cycle, and maintained on a commercial pelleted chow diet (PROVIMI KLIBA SA, Switzerland) consisting, by energy, of 24% protein, 66% carbohydrate, and 10% fat, and had free access to tap water. Animals were maintained in accordance with the regulations and guidelines of the Department of Medicine, University of Fribourg, for the care and use of laboratory animals; all experimental procedures were performed under conditions approved by the Ethical Committee of the State of Fribourg Veterinary Office. Hyperinsulinemic-euglycemic clamps were conducted at the University of Geneva according to the procedures approved by the animal care and experimentation authorities of the Canton of Geneva, Switzerland.

### Experimental design

The design was similar as previously reported using our rat model of semistarvation, with the rats studied being in an age range characterized by a high rate of weight gain during spontaneous growth^7,11^. During the food restriction (semistarvation) period, the animals were fed a fixed ration diet of 14 g chow daily represented ~50% of their spontaneous ad libitum daily food intake. After 2 weeks of semistarvation that induced growth arrest, the animals with body weight in the range of 230–250 g were distributed into three groups (*n* = 8–9) such that each group had similar mean body weight and standard deviations, and was provided (non-blinded) with an isocaloric amount of one of three different diets: a low-fat (LF) diet or two high fat (HF) diets made either of lard, i.e. low in polyunsaturated fatty acids and high in saturated and monosaturated fatty acids (HF-SMFA diet), or a HF diet rich in essential polyunsaturated fatty acids (HF-ePUFA diet), the fat source consisting of a 1:1 mixture of safflower oil and linseed oil; the nutrient composition of these diets is provided as supplementary information (SI 1); the details of fatty composition analysis and measurements of metabolizable energy (ME) intake have been reported previously^11,12^. All groups were thus provided with the same amount of ME intake (355 kJ/day/rat), which corresponds to that consumed during spontaneous food intake on pelleted chow; each refed rat consumed all the food provided on a daily basis throughout the entire refeeding period. The level of fat in the HF diets (58% of energy intake) corresponds to dietary fat levels often utilized in rehabilitation (energy-dense) diets of malnourished infants and children in order to meet their high-energy requirements for catch-up growth^13,14^. Four experiments semistarvation–refeeding of similar design were conducted with these three diets refed isocalorically. Experiment I was conducted to assess energy balance and body composition over 2 weeks of refeeding, with a test of glucose tolerance conducted on days 7–8. Experiments II and III were two insulin clamp studies (with high and low dose of insulin), each clamp study was performed after 7–8 days of refeeding. The last experiment (IV) was conducted to harvest adipose tissues for assessing key de-novo lipogenic enzymes after one week of refeeding on these three diets.

### Body composition analysis

After the animals were sacrificed, the whole carcasses were dried to a constant weight in an oven maintained at 70 °C and were subsequently homogenized for analysis of total fat content by the Soxhlet extraction method^15^. Body water was calculated as the difference between body weight before and after drying, and the dry lean mass was determined by subtracting total body fat and body water content from body weight.

### Glucose tolerance test

Glucose tolerance tests were performed on days 7 and 8 of refeeding, according to the protocol described previously^7,9^. Food was removed early in the morning (07:00 h). At 6–7 h later, i.e. in the post-absorptive phase, blood was drained from the tail vein and immediately followed by an intraperitoneal injection of glucose (2 g/kg body weight). At intervals of 30 min for the next 2 h period, blood samples were taken from the tail vein in heparinized tubes and transferred on ice. The blood samples were then centrifuged, and the plasma was frozen and stored at −20 °C for later assays of plasma glucose and insulin. Plasma glucose was determined using a Beckman Glucose Analyzer (Beckman Instruments, Palo Alto, CA, USA), while plasma insulin was assessed using a rat insulin ELISA kit (Crystal Chem, Inc., Downer’s Grove, IL, USA).

### Hyperinsulinemic-euglycemic clamp

On the day of insulin clamp experiments (day 7 or 8 of refeeding), rats were anesthetized (09:00 h) with Nembutal^®^ (i.p., 50 mg/kg; Abbott Laboratories, Chicago, IL, USA). Surgeries were performed as previously described in detail^16,17^ and body temperature of rats was maintained at 37 °C throughout the study by means of a heating blanket. Glucose infusion rate (GIR) to maintain euglycemia was measured under basal and insulin-stimulated (200 mU/mL Actrapid^®^ HM, Novo Nordisk, Bagsvaerd, Denmark) conditions, as previously described^16,17^. Insulin was infused at a dose (18 mUI/kg/min) known to ensure complete suppression of hepatic glucose production^16^. In another separate insulin clamp experiment conducted at the same time-point of refeeding (day 7–8), the dose of insulin infused was halved (9 mUI/kg/min) such that plasma insulin could be clamped at values corresponding to peak plasma insulin values (12–15 ng/mL) previously found after a glucose load in this rat model showing catch-up fat^7,9^.

At the end of the insulin clamps, the in vivo insulin-stimulated glucose utilization index of individual tissues was determined using 2-deoxy-d-[1-3H] glucose (30 µCi/rat; Amersham Biosciences UK Ltd, Buckinghamshire, UK). A bolus of 2-deoxy-glucose was injected through the jugular vein and aliquots of arterial samples were regularly collected. After 30 min, rats were killed by decapitation and tissues rapidly removed and stored at –80 °C. The 2-deoxy-d-[1-3H] glucose-specific activity was determined in ZnSO_4_ and Ba(OH)_2_ deproteinized blood samples^16,17^. Measurement of tissue concentration of 2-deoxy-d-[1-3H]glucose-6-phosphate allowed calculation of the in vivo glucose utilization index of individual tissues, and was expressed in ng/min/mg of tissue. Plasma glucose and insulin levels were determined under basal and clamps conditions by the glucose oxidase method (Glu, Roche Diagnostics GmbH, Rotkreuz, Switzerland) and enzyme immunoassay (SPI bio, Montigny Le Bretonneux, France), respectively.

### Assay of de-novo lipogenic enzyme activities

Fatty acid synthase (FAS) and glucose-6-phosphate dehydrogenase (G6PDH) activities were measured as previously reported^9^; the details are provided as supplementary information (SI 2).

### Data analysis and statistics

All rats were included in the analysis; none satisfied the pre-determined exclusion criteria of abnormal behavior such as hyperactivity or lack of food intake. A sample size of a minimum of seven animals per diet group was based on previous experiments that detected significant differences in key parameters of body composition and glucose homeostasis between low-fat and high-fat diets^9^. All data are presented as means with their standard errors. The data was analyzed by one-way analysis of variance (ANOVA), followed by post-hoc pairwise comparisons using Scheffe’s test across the three diet groups; and differences were considered to be statistically significant at *p* < 0.05. The statistical treatment of data was performed using the computer software STATISTIX, version 8.0 (Analytical Software, St. Paul, MN, USA). Statistical tests and group sizes are specified in the table legends.

## Results

### Body composition

**Fig. 1:**
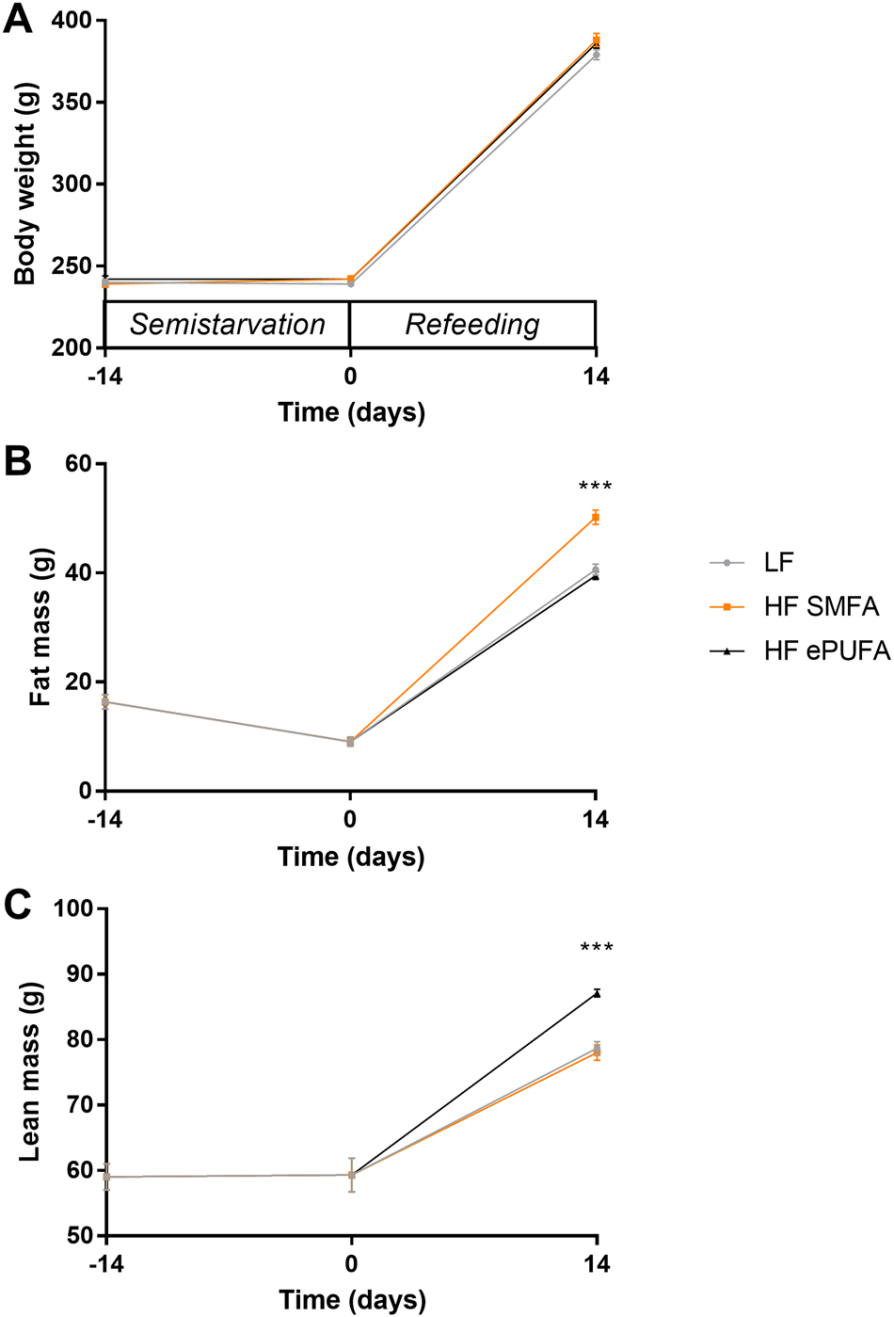
Profile of body weight and body composition in response to semistarvation and refeeding. Body weight (**A**), fat mass (**B**), and dry lean mass (**C**) at the beginning (day −14) and end of semistarvation (day 0), and after 2 weeks of isocaloric refeeding (day 14); the refed diets are as follows: LF low fat, HF SMFA high-fat saturated monounsaturated fatty acids, HF ePUFA high-fat essential polyunsaturated fatty acids. Values are means±SE (*n* = 7–8). Data are analyzed by one-way analysis of variance (***p < 0.001).

The body weight and body composition profiles of the groups are shown in Fig. 1. Semistarvation resulted in growth arrest (panel A), such that relative to their weights at the onset of the food restriction period (Day −14), the body weight of the food-restricted rats (about 240 g on average) was only slightly reduced at the end of semistarvation (Day 0). Comparison of the body composition of groups of animals at the onset and at the end of the 2-week semistarvation period shows significant reductions (of about 45%) in body fat but not in lean mass (panels B and C). During isocaloric refeeding over a period of 2 weeks, animals refed with the HF-SMFA diet showed significantly greater gain in body fat resulting in significantly higher fat mass (+25%, *p* < 0.001), but similar gain in dry lean mass than animals refed the LF diet. The HF-ePUFA refed group, by contrast, showed significantly less gain in body fat but more gain in lean mass than those refed on the HF-SMFA diet. Thus, the HF-ePUFA diet abolished the differences in body fat between the HF-SMFA and LF groups, and resulting in a higher lean mass relative to the two other groups.

### Glucose tolerance test

**Fig. 2:**
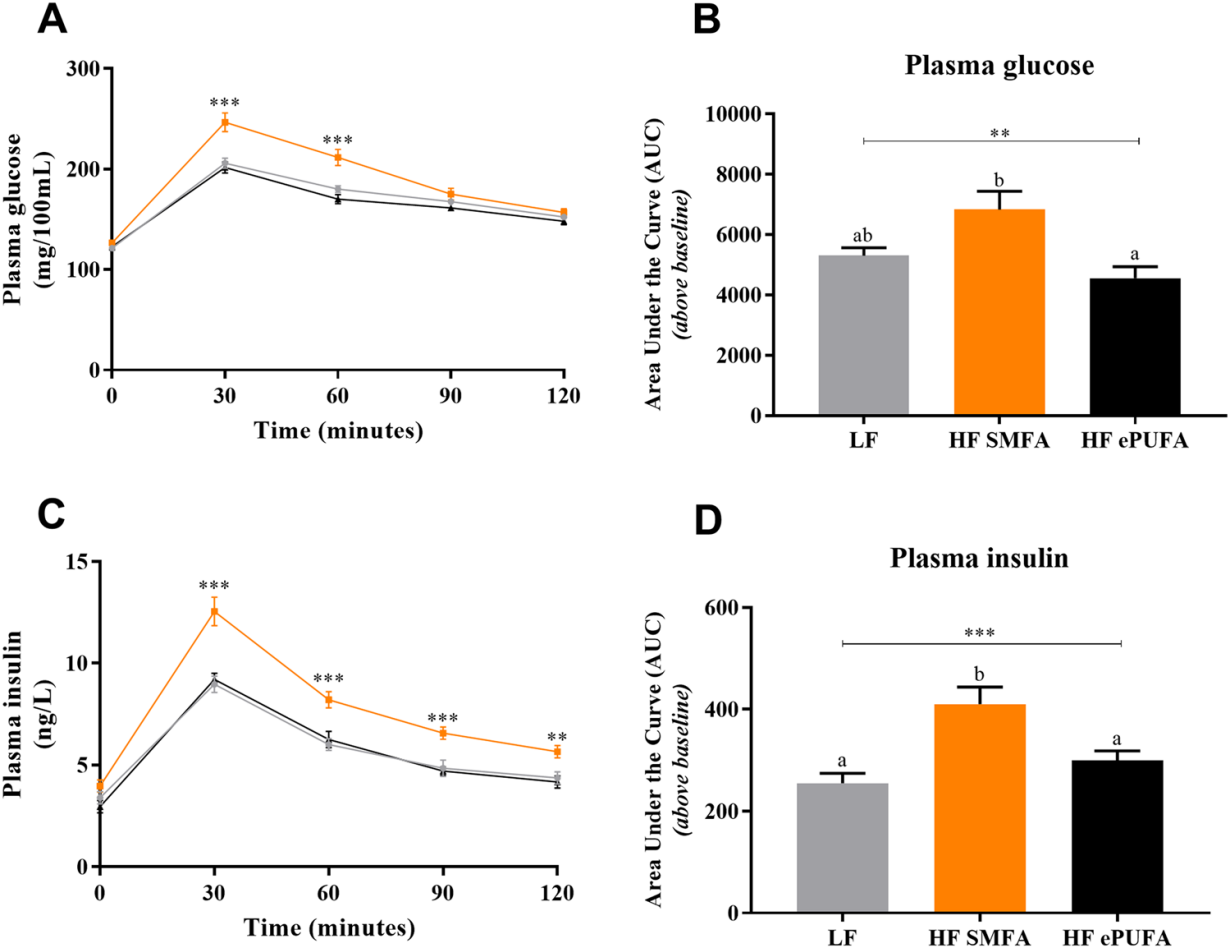
Glucose tolerance test in animals refed for 7–8 days on a low-fat or high-fat diets. Plasma glucose and insulin response curves after glucose administration (panel **A** and **C**, respectively) and related area-under-the-curve for glucose and insulin (panel **B** and **D**, respectively). LF low fat, HF SMFA high-fat saturated monounsaturated fatty acids, HF ePUFA high-fat essential polyunsaturated fatty acids. All values are means ± SE (*n* = 8). Data are analyzed by one-way analysis of variance (ANOVA) (**p < 0.01, ***p < 0.001), followed by post hoc pairwise comparisons using Scheffe test. Values not sharing a common superscript letter are significantly different at *p* < 0.05.

The results of the glucose tolerance test, conducted on days 7–8 of refeeding, are shown in Fig. 2. No differences are observed in basal post-absorptive plasma glucose (time-point 0) among the three groups (panel A). After glucose administration, the glucose response curve was higher in the HF-SMFA group than in the LF or HF-ePUFA groups over the first hour (panel A) with these differences reflected in the area-under-the-curve for plasma glucose being higher in the HF-SMFA group than in the LF or HF-ePUFA groups but not between the LF and HF-ePUFA groups (panel B). The results for plasma insulin (Fig. 2, panels C and D) indicate no significant differences in basal (post-absorptive) plasma insulin, although the value for the HF-ePUFA group tended to be lower than in the other two groups (panel C). Following the administration of glucose, the plasma insulin response curve in HF-SMFA group was higher than in the LF or HF-ePUFA groups, with significant differences when assessed as area-under-the-curve for plasma insulin between HF-SMFA group and the LF or HF-ePUFA groups, but not between the LF and HF-ePUFA groups (panel D).

### In-vivo glucose utilization during hyperinsulinemic-euglycemic clamps

Table 1 shows the results of the two separate insulin clamp experiments, conducted with exactly the same design, but with the second experiment involving the administration of about half the dose of insulin during the clamp than in the first experiment; these two experiments are hence referred to a high-dose clamp and low-dose clamp, respectively. In both clamp experiments, there was no significant differences in the basal (pre-clamp) plasma glucose concentrations across the three diet groups. By contrast, the basal insulin concentrations were significantly different across diets (*p* < 0.01), with the value for the HF-ePUFA group being lower than in the HF-SMFA by ~24% and 18% in the high-dose and low-dose clamp experiments, respectively. In both clamp experiments, there was also a marked difference across diets for the glucose infusion rate (GIR) with the two groups refed high-fat diets showing 25–50% lower GIR than in the LF group (*p* < 0.001). Relative to animals refed the LF diet, those refed the HF-SMFA and HF-ePUFA diets showed a 48% and 47% reduction in the GIR, respectively, in the high-dose clamp study, and a 32% and 23% reduction in the GIR, respectively, in the low-dose clamp study. Direct comparison between the two high-fat diets indicate a tendency for a higher GIR in the HF-ePUFA group than in the HF-SMFA group, particularly in the low-dose clamp study; however, no significant differences could be detected by a post-hoc pair-wise comparison test after ANOVA.

**Table 1:** Metabolic parameters during hyperinsulinemic-euglycemic clamps at two doses of insulin (high and low) in groups of rats refed isocalorically on either a low-fat (LF) diet, a high-fat diet rich in saturated and monounsaturated fat (HF SMFA), or a high fat diet rich in essential polyunsaturated fat (HF ePUFA) for 7–8 days.

	LF	HF SMFA	HF ePUFA	ANOVA
**I.** *Insulin clamp (high dose)*				
Plasma glucose (mg/100 mL)				
Basal	117 ± 3	105 ± 3	111 ± 6	NS
Insulin stimulated	120 ± 3	124 ± 3	126 ± 3	NS
Plasma insulin (ng/mL)				
Basal	3.08 ± 0.09^a^	2.87 ± 0.18^a^	2.19 ± 0.15^b^	*p* < 0.01
Insulin stimulated	29.6 ± 1.2^ab^	32.9 ± 1.3^a^	27.7 ± 1.1^b^	*p* < 0.05
Glucose infusion rate (mg/min/kg)	35.4 ± 0.9^a^	18.4 ± 0.6^b^	18.9 ± 0.6^b^	*p* < 0.001
**II**. *Insulin clamp (low dose)*				
Plasma glucose (mg/100 mL)				
Basal	116 ± 5	109 ± 6	112 ± 9	NS
Insulin stimulated	128 ± 8	128 ± 4	132 ± 9	NS
Plasma insulin (ng/mL)				
Basal	3.02 ± 1.10^a^	2.15 ± 0.66^ab^	1.76 ± 0.35^b^	*p* < 0.01
Insulin stimulated	14.9 ± 5.5	12.6 ± 2.3	13.5 ± 4.4	NS
Glucose infusion rate (mg/min/kg)	31.0 ± 3.6^a^	21.1 ± 1.2^b^	23.8 ± 2.4^b^	*p* < 0.001

Data are analyzed by one-way analysis of variance (ANOVA) followed by post hoc pairwise comparisons using Scheffe’s test. Values not sharing a common superscript letter are significantly different at *p* < 0.05. NS: not statistically significant. Values are mean ± SE (*n* = 7–9).

**Fig. 3:**
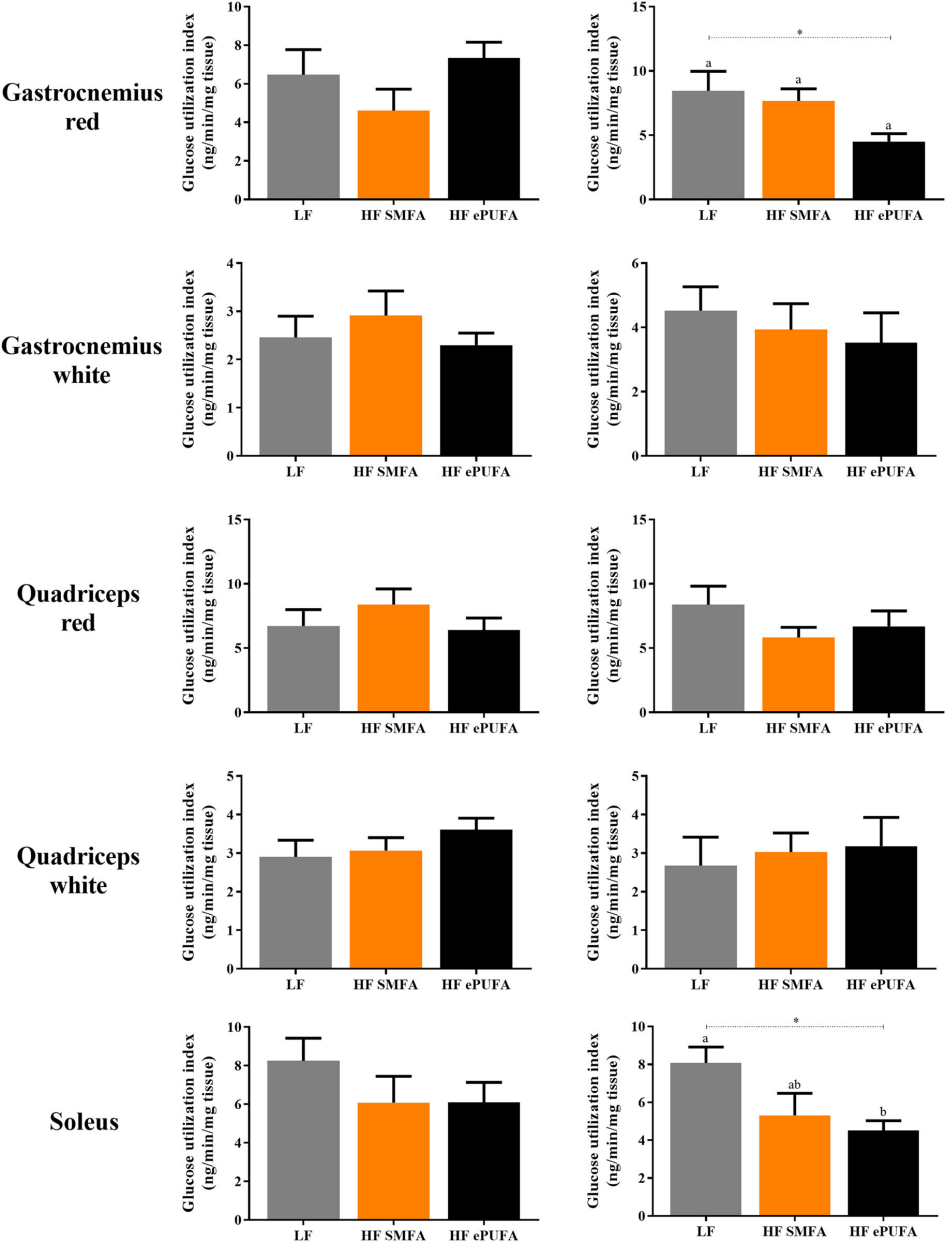
Glucose utilization index (GUI) of skeletal muscles under a high (18 mUI/kg/min) (left panel) and a low (9 mUI/kg/min) (right panel) insulin dose during hyperinsulinemic-euglycemic clamp at day 7–8 of refed on a low-fat or high-fat diets. LF low fat, HF SMFA high-fat saturated monounsaturated fatty acids, HF ePUFA high-fat essential polyunsaturated fatty acids. Values are means ± SE (*n* = 7–9). Data are analyzed by one-way analysis of variance (ANOVA) (*p < 0.05) followed by post hoc pairwise comparisons using Scheffe’s test. Values not sharing a common superscript letter are significantly different at *p* < 0.05.

**Fig. 4:**
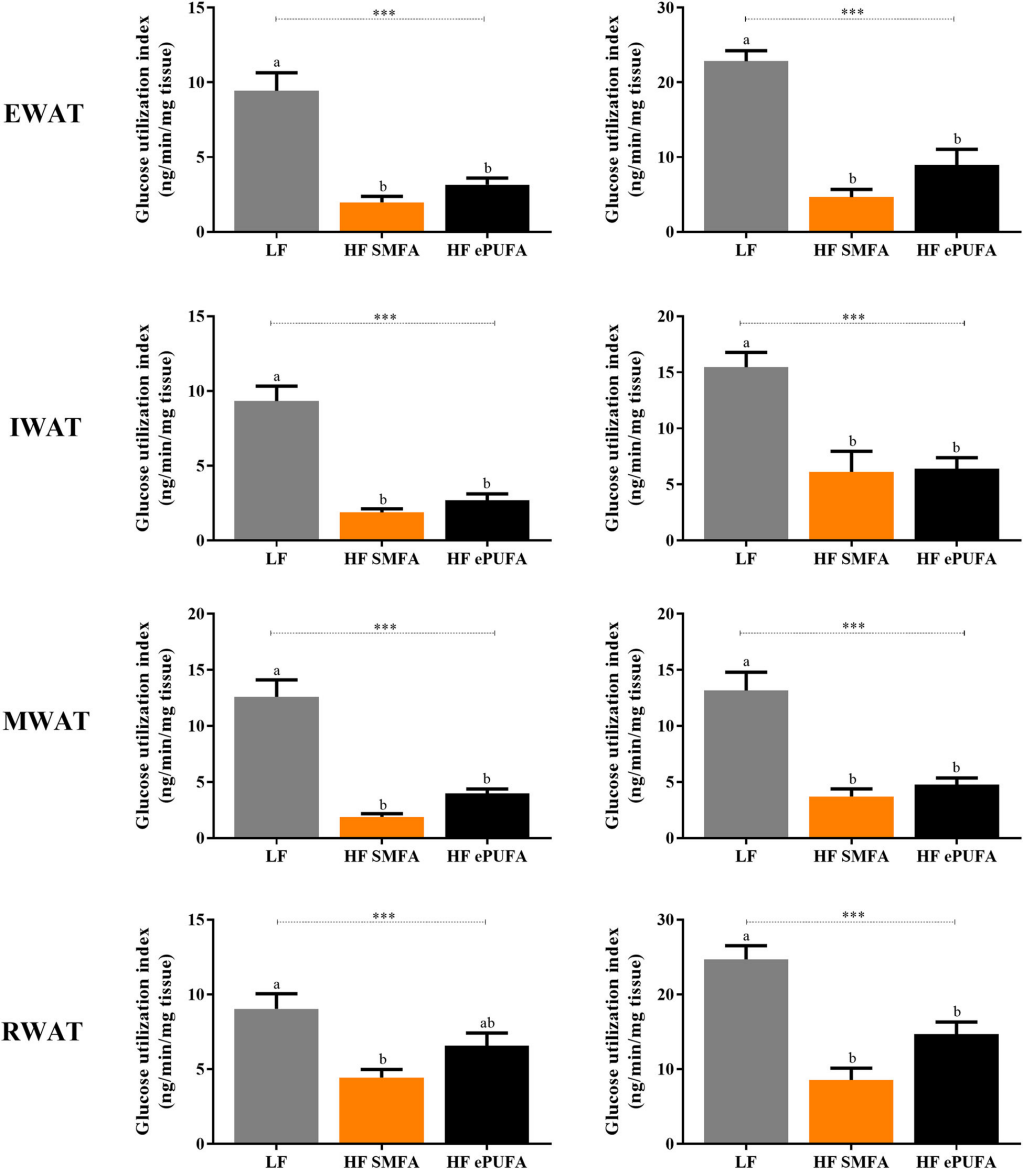
Glucose utilization index (GUI) of white adipose tissues (WAT) under a high (18 mUI/kg/min) (left panel) and a low (9 mUI/kg/min) (right panel) insulin dose during hyperinsulinemic-euglycemic clamp at day 7–8 of refed on a low-fat or high-fat diets. LF low fat, HF SMFA high-fat saturated monounsaturated fatty acids, HF ePUFA high-fat essential polyunsaturated fatty acids, EWAT epididymal white adipose tissue, IWAT inguinal white adipose tissue, MWAT mesenteric white adipose tissue, RWAT retroperitoneal white adipose tissue. Values are means ± SE (*n* = 7-9). Data are analyzed by one-way analysis of variance (ANOVA) (***p < 0.001) followed by post hoc pairwise comparisons using Scheffe’s test. Values not sharing a common superscript letter are significantly different at *p* < 0.05.

The data on tissue-specific insulin-stimulated glucose utilization index (GUI) are presented in Fig. 3 for skeletal muscle and in Fig. 4 for adipose tissue; in both these figures, the left panels providing data for the high-dose insulin clamp experiment and the right panels providing data from the low-dose insulin clamp experiment. There are no significant differences across the three diets for GUI in all skeletal muscles studied in the high dose insulin clamp experiment (Fig. 3, left panel). Similarly in the low-dose insulin clamp experiment, GUI was not significantly different in the groups refed the three diets in skeletal muscle (Fig. 3, right panel), except in the red gastrocnemius and soleus muscles, where GUI was lower in the group refed the HF-PUFA diet compared to the one refed the LF diet. However, these latter differences were of borderline statistical significance, so that the possibility of a false positive cannot be disregarded. Furthermore, no significant differences are observed between the two high-fat refed groups (HF-ePUFA vs. HF-SMFA) for GUI in any of the six muscles studied.

The data on GUI in adipose tissues indicate highly significant differences across diets for all adipose tissue depots studied (Fig. 4, left and right panels), with GUI being several folds lower in the two high-fat groups than in the LF group, and post-hoc multiple pairwise comparison tests after ANOVA indicating significant differences between the LF and HF diets, but not between the two HF diets. There is, however, a tendency for GUI in adipose tissues from HF-ePUFA group to be higher than those from the HF-SMFA group, particularly in epididymal (EWAT) and retroperitoneal (RWAT) WAT where direct comparison by unpaired *t*-test shows borderline statistical significance.

### De-novo lipogenic enzyme activities

**Fig. 5:**
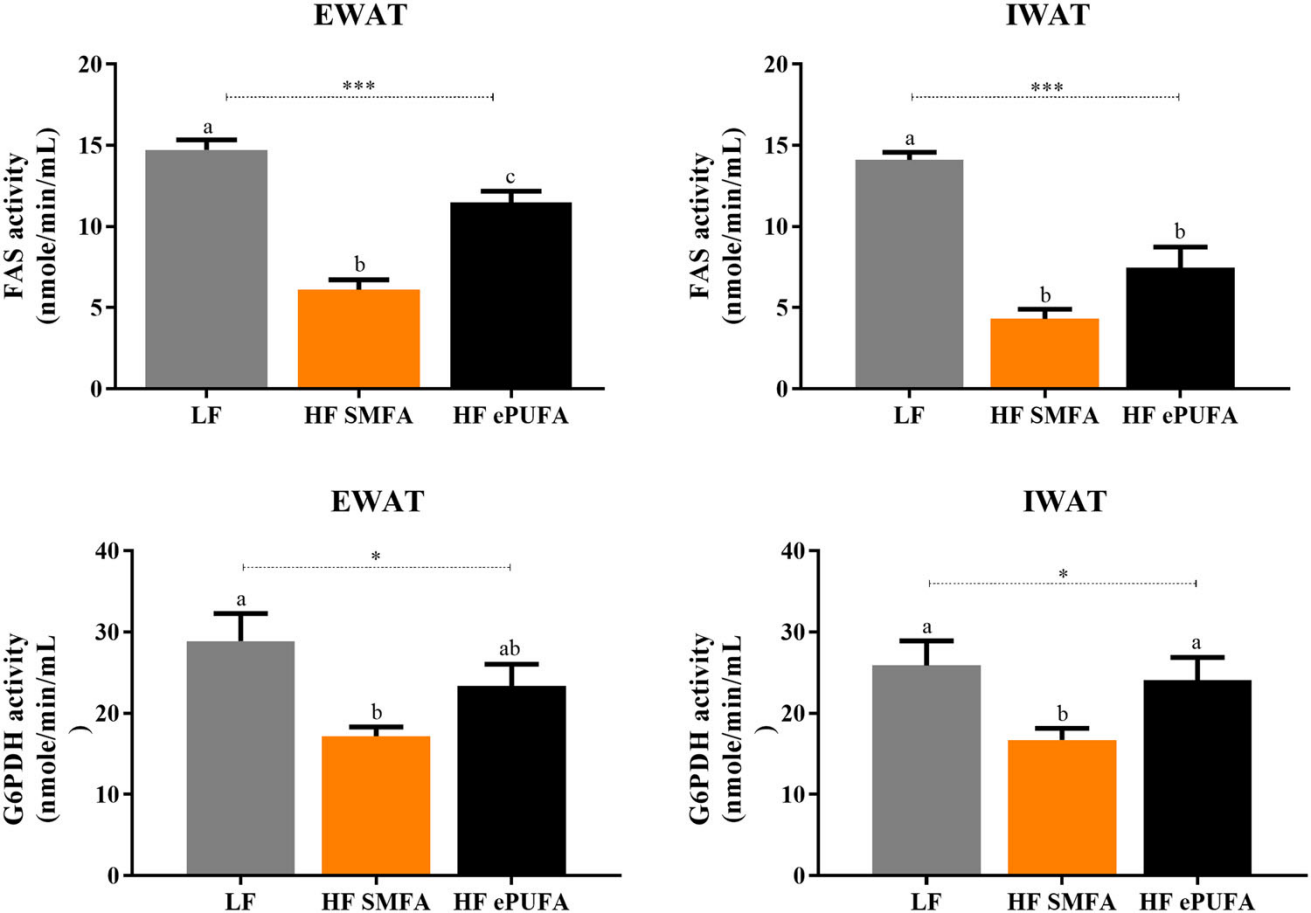
De-novo lipogenic enzyme activities (FAS and G6PDH) in two white adipose depots (EWAT and IWAT) from rats refed with a low-fat or high-fat diets for a week. LF low fat, HF SMFA high-fat saturated monounsaturated fatty acids, HF ePUFA high-fat essential polyunsaturated fatty acids, EWAT epididymal white adipose tissue, IWAT inguinal white adipose tissue. Values are means ± SE (*n* = 7–8). Data are analyzed by one-way analysis of variance (ANOVA) (*p < 0.05, ***p < 0.001) followed by post hoc pairwise comparisons using Scheffe’s test. Values not sharing a common superscript letter are significantly different at *p* < 0.05.

The results of FAS and G6PDH activities in adipose tissues (EWAT and inguinal WAT) are shown in Fig. 5. In EWAT, the activity of FAS on the HF-SMFA diet is found to be lower than on LF diet by about 59% (*p* < 0.001), but on the HF-ePUFA, this reduction relative to the LF diet is less pronounced (about 21%, *p* < 0.001). Concerning G6PDH activity in EWAT, we observe the same tendency, namely a decrease of 41% on HF-SMFA diet and 19% for HF-ePUFA diet, with ANOVA analysis indicating significant difference across diets (*p* < 0.05). In IWAT, comparisons across all three diets indicate that FAS activity is significantly decreased on HF-SMFA and HF-ePUFA diets relative to LF rats (−70% and −47%, respectively, *p* < 0.001), and there is a tendency for FAS activity to be higher on the HF-ePUFA diet than on the HF-SMFA diet. For G6PDH activity in IWAT, a significant decrease is observed in HF-SMFA rats relative to LF rats (−36%, *p* < 0.05), but not in HF-ePUFA rats. Overall, the reduction in activity of these two key de novo lipogenic enzymes in the adipose tissues of rats refed the high fat diets (relative to the LF diet) is less pronounced during refeeding on the high ePUFA diet.

## Discussion

Previous studies in this rat model of semistarvation-refeeding have demonstrated that compared to spontaneous growing control animals, the efficiency of fat deposition during refeeding is increased on a low fat diet and is accompanied by an early development of hyperinsulinemia, skeletal muscle insulin resistance but increased adipose tissue insulin sensitivity, as assessed by the insulin clamp technique^7,8^. Isocaloric refeeding on a typical ‘western’ high fat (HF-SMFA) diet results in an even higher efficiency of fat deposition, exacerbated hyperinsulinemia^7,9^, persistent skeletal muscle resistance but blunting of adipose tissue insulin hyper-responsiveness pertaining to glucose utilization^9^. However, this exacerbation of the efficiency of fat deposition and excessive hyperinsulinemia observed with the HF-SMFA diet could be prevented by isocaloric refeeding on diets rich in ePUFA^10–12^. In our study here, we confirm the latter demonstrations and furthermore show that, contrary to our hypothesis, the efficacy of the e-PUFA diet in countering the impaired glucose homeostasis resulting from the HF-SMFA diet, does not reside in increased insulin sensitivity in skeletal muscle and is only marginally related to improved insulin sensitivity in adipose tissue.

### Whole-body insulin sensitivity

Examination of the data on plasma glucose and insulin in the basal post-absorptive state (i.e. prior to the test of glucose tolerance or prior to the insulin clamp studies) indicate that although basal plasma glucose was similar across the three diet groups, basal plasma insulin, however, tended to be lower in the ePUFA group. While a lower basal insulin requirement for maintaining basal glucose levels may be interpreted as being consistent with a state of higher whole-body insulin sensitivity, this remains merely an association and does not provide direct proof for a higher whole-body insulin sensitivity. Using the hyperinsulinemic-euglycemic clamp, which is widely considered the gold standard method for assessing insulin action in vivo, we found the glucose infusion rate (GIR) to be markedly lower in both HF groups than in the LF group. Although GIR tended to be higher in the HF-ePUFA group than in the HF-SMFA group, this difference was small and not statistically significant. Thus, the ability of the HF-ePUFA diet to lower basal insulin and to counter the higher insulin and glucose responses observed with the HF-SMFA diet during the test of glucose tolerance is not reflected in a significant improvement in whole-body insulin sensitivity assessed during the insulin clamps.

### Tissue-specific insulin sensitivity

The examination of data on tissue-specific insulin-stimulated glucose utilization index (GUI), assessed by the additional use of labeled 2-deoxyglucose at the end of the clamps, revealed that there is no increase in insulin sensitivity in skeletal muscle by the high ePUFA diet. Indeed, animals refed the HF-ePUFA diet did not show an increase in GUI in any of the six different skeletal muscles studied, thereby underscoring the fact that this lack of increase in muscle insulin sensitivity by HF-ePUFA refeeding is independent of skeletal muscle type and fiber composition. By contrast, our results in WAT may be interpreted as providing partial support to our hypothesis that the HF-ePUFA diet increases insulin sensitivity in adipose tissue, given the tendency for GUI to be higher than in WAT from animals refed the HF-ePUFA diet than on the HF-SMFA diet. These latter findings would be consistent with the tendency for a higher activity of key de novo lipogenic enzymes (FAS and G6PDH) in tissues from the HF-ePUFA than in the HF-SMFA group as demonstrated here in EWAT and IWAT, and also as previously reported by Crescenzo et al. in EWAT^12^. However, the improvement in GUI in all WAT depots studied here is very modest as the HF-ePUFA diet only minimally prevented the marked drop in adipose tissue GUI observed in the HF-SMFA group relative to the LF group. Furthermore, the higher GUI in adipose tissues from animals refed the HF-ePUFA than in those refed the HF-SMFA diet is found to be either of borderline statistical significance or failed to achieve statistical significance in the WAT depots studied.

### Beyond insulin-dependent mechanisms for improved glucose homeostasis

Taken together, the results presented here suggest that while refeeding on the high ePUFA diet reduces basal plasma insulin and prevents the higher insulin and glucose response curve to a glucose load found during refeeding on the HF-SMFA diet, these improvements in glucose homeostasis are unlikely to be primarily attributed to an increase in insulin action on glucose uptake and utilization, as evidenced in the skeletal muscles, adipose tissues, and at the whole-body level. Consequently, the question arises as to what could be the other mechanisms in these or other tissues that may dispose of glucose, and account for the major impact of refeeding HF-ePUFA diet in reducing hyperinsulinemia and allowing the achievement of blood glucose homeostasis to that found in low-fat refed animals.

One explanation may reside in insulin-independent mechanisms by which high levels of ePUFA may stimulate glucose uptake and utilization in the skeletal muscle and other tissues. In fact, the existence of insulin-independent pathways for glucose uptake in skeletal muscle has long been known^18^. The contraction of skeletal muscle in vitro increases glucose transport in the absence of insulin^19^, and insulin and muscle contraction stimulate the translocation of GLUT4 glucose transporters to the cell membrane (and consequently glucose uptake) through distinct pathways^20^. Among the signaling factors that may mediate pathways for glucose uptake in contracting skeletal muscle are the activation of AMP-activated protein kinase (AMPK)^21,22^, calcium concentrations^23^, nitric oxide^24,25^, and reactive oxygen species^26,27^, several of which have also been implicated in muscle glucose uptake independently of both insulin and muscle contraction^18^. They can be modulated by the neuroendocrine system (including sympathetic nervous system, adiponectin, leptin, and thyroid hormones), and could also be involved in the mechanisms by which ePUFA may influence the regulation of glucose and fatty acid metabolism in peripheral tissues. Such a contention would be consistent with the effects of diets enriched in n-3 PUFA in the prevention of weight gain and insulin resistance^28^, and which are thought to be mediated by adiponectin and leptin, two adipokines that regulate glucose and lipid metabolism, through AMPK activation^29–31^. Furthermore, the direct effects of leptin on skeletal muscle thermogenesis have been shown to require glucose entry through insulin-independent mechanisms that require intact phosphatidylinositol 3-kinase and AMPK signaling^32^.

Whether one or more of these factors implicated in insulin-independent glucose uptake in response to muscle contraction or to leptin and adiponectin in muscle or other tissues may be stimulated by ePUFA is not known, but PUFAs are known to regulate the expression of genes encoding proteins involved in the regulation of energy metabolism by acting as agonist ligands of peroxisome proliferator-activated receptors (PPARs)^33^. In particular, there is some evidence that PUFA may serve as a natural regulator of glucose uptake in vivo and these effects are mainly through PPARγ function^34^. Furthermore, isomers of conjugated linoleic acids have been shown to act on pathways that are both insulin-dependent and independent via PPARs, and that they can mimic insulin action by stimulating glucose uptake and GLUT4 trafficking through isomer-specific effects in stimulating PI3-kinase or AMPK signaling^35^.

Finally, it should be pointed out that the effects of ePUFA in diets based on safflower and linseed oils (as utilized here) might be mediated via their essential fatty acids (linoleic acid and α-linolenic acid) per se as well as via their elongated-desaturated products such as arachidonic acid, docosahexaenoic acid, and eicosapentaenoic acid. Whether the non-insulin-dependent pathways by which ePUFA (linoleic or linolenic acids) themselves or their metabolites are also involved in the efficacy of the HF-ePUFA diets to increase lean mass as shown here and previously is unknown, but a higher lean mass in its own right would increase the glucose buffering capacity and glucose clearance and hence contribute to improving glucose homeostasis.

In conclusion, our study indicates that during refeeding on the high-fat rich in ePUFA, in vivo assessed insulin sensitivity pertaining to glucose utilization is increased only marginally in adipose tissues, and not at all in skeletal muscle, resulting in a marginal improvement in whole-body insulin sensitivity. It is argued on the basis of these findings that the efficacy of ePUFA in improving glucose homeostasis is only marginally contributed by increased insulin sensitivity, and that the major contribution to glucose disposal and blood glucose homeostasis during refeeding on a high ePUFA diet could reside in (i) insulin-independent mechanisms that stimulates glucose entry in skeletal muscle and other tissues and (ii) in increased protein retention in skeletal muscle and in other organs/tissues, thus providing an increased lean mass effect as glucose buffering capacity.

## Supplementary information

Supplementary materials

## Data Availability

The datasets generated during the current study are available from the corresponding author on reasonable request.
