# Supplementary material for: Countering impaired glucose homeostasis during catch-up growth with essential polyunsaturated fatty acids: is there a major role for improved insulin sensitivity?
Source: Nutr Diabetes. 2021 Jan 7;11:4. doi: 10.1038/s41387-020-00143-y (PMC7791023; doi:10.1038/s41387-020-00143-y)
Supplement: Supplementary file 1 — Supplementary materials [file 41387_2020_143_MOESM1_ESM.docx]

*Supplementary Material S1*

**S1. Composition of high-fat (HF) diets rich in saturated-monounsaturated (SFA-MUFA) or in essential polyunsaturated fatty acids (ePUFA).**

**HF SMFA HF ePUFA**

**Components** (*g/100g diet)* ^‡^

Basal mix*

Chow 59.0 59.0

Casein 10.8 10.8

Methionine 0.1 0.1

Choline chloride 0.1 0.1

Vitamin mix 0.5 0.5

Mineral mix 1.7 1.7

Sunflower oil 1.4 1.4

Added test fat/oils^†^

Lard 26.0 -

Safflower oil - 13

Linseed oil - 13

**Metabolisable energy (ME)** *(kJ/g)*^§^ 19.6 19.6

**Macronutrients** *(% ME)*

Protein 21.0 21.0

Lipid 58.2 58.2

Carbohydrate 20.8 20.8

**Fatty acid composition** *(%ME)*

Saturated fatty acids 25.1 6.0

Monounsaturated fatty acids 24.8 10.6

Polyunsaturated fatty acids (PUFA) 8.2 41.6

n-6 PUFA *(linoleic acid)*  7.3 28.5

n-3 PUFA *(α linolenic acid)*  0.9 13.1

^*^ The basal mix provides 50% of total energy content, and its fat content contributes 8.2 % of energy content (1.5% from saturated fatty acids (SFA), 2.6% as monounsaturated fatty acids (MUFA), 4.1% as polyunsaturated fatty acids (PUFA)). The food ingredients were purchased as follows: ground standard chow (Provimi-Lacta, Switzerland); sunflower oil (Migros, Switzerland); vitamin-free casein, DL methionine, choline chloride (Sigma, St Louis, MO); AIN 76 vitamin mixture & AIN 76 mineral mixture from MP Biomedicals (Ohio, USA).

^†^ The added fat was either lard (control diet) or test oils rich in ePUFA made up of a 1:1 mix of safflower and linseed oils; these high ePUFA oils which were purchased from MP Biomedicals (Ohio, USA). For the low- fat diet, the added fat (26g) was replaced isocalorically with sugars (58.5g) consisting of a 1:1 mix of sucrose and glucose.

^‡^ The diet ingredients were purchased from companies in Switzerland: casein (Schweizerhall); L-cystein (Fluka); AIN-93 M mineral mix and AIN-93 M vitamin mix (Socochim); sucrose (Howeg); safflower oil (Sofinol SA, Manno); linseed oil (Sabo, Manno); choline bitartrate was purchased from Synopharm (Germany).

^§^ Metabolisable energy (ME) density was estimated by computation using values (kJ/g) for ME content of chow, 13.0; fat/oil, 37.6; carbohydrates, 16.7; protein, 16.7.

*Supplementary Material S2*

**S2: Assay of de-novo lipogenic enzyme activities**

Fatty acid synthase (FAS) and Glucose-6-Phosphate deshydrogenase (G6PDH) activities were assessed as previously reported (1), and according to a modified method of Pénicaud et al (2). Tissue samples (200 mg) were first homogenized in 600 µL of 0.25M ice-cold sucrose solution containing EDTA (1 mM), dithiothreitol (DTT, 1 mM), proteases inhibitors (Complete®, Sigma-Aldrich, St-Louis, MO, USA), and adjusted at pH 7,4. Mixtures were centrifuged at 12000 g during 10 minutes at + 4°C. The resulting supernatants containing cytosolic proteins were collected and assayed by NanoDrop system (Thermo Fisher Scientific, Wilmington, DE, USA). For FAS activity, these extracts were assayed using 50 μl of extract, and using a spectrophotometer set at 340 nm at 37°C. The readings were performed by sequentially adding the extract into the cuvettes, followed by 150 μl of solution containing 61.7 mM of acetyl-CoA and 0.15 mM of NADPH in FAS buffer (K-phosphate stock solution, pH 6.5), and followed by 30 μl of 8 mg/ml malonyl-CoA. For G6PDH activity, these extracts were assayed using 20 μl of extract, and using a spectrophotometer set at 340 nm at 37°C. The readings were performed by sequentially adding the extract into the cuvettes, followed by 100 μl of 0.2 M G-6-P solution in glycyl-glycin buffer (0.25 M, pH 7,6), and 10 μl of NADP. Then, FAS and G6PDH activities expressed per unit of cytosolic proteins.

**References**

1. Marcelino H, Veyrat-Durebex C, Summermatter S, Sarafian D, Miles-Chan J, Arsenijevic D et al. A role for adipose tissue de novo lipogenesis in glucose homeostasis during catch-up growth: a Randle cycle favoring fat storage. Diabetes. 2013;62:362-72.

2. Pénicaud L., Ferré P., Assimacopoulos-Jeannet F., Perdereau D., Leturque A., Jeanrenaud B., Picon L., Girard J. Increased Gene Expression of Lipogenic Enzymes and Glucose Transporter in White Adipose Tissue of Suckling and Weaned Obese Zucker Rats. Biochem. J. 1991; 279: 303–308.
